# Prevalence of Hepatitis B Virus, Genotypes, and Mutants in HBsAg-Positive Patients in Meerut, India

**DOI:** 10.29252/.23.5.354

**Published:** 2019-09

**Authors:** Salman Khan, Molly Madan, Sunil Kumar Virmani

**Affiliations:** 1 Department of Microbiology, Netaji Subhash Chandra Bose Subharti Medical College, Swami Vivekanand Subharti University, Meerut, Uttar Pradesh, India;; 2 Department of Medicine, Netaji Subhash Chandra Bose Subharti Medical College, Swami Vivekanand Subharti University, Meerut, Uttar Pradesh, India

## Abstract

**Background:**

Genetic changeability of hepatitis B virus (HBV) signifies a challenge for the sensitivity of immunologic and molecular diagnostics. Therefore, knowing the spread of HBV genotypes (GENs) and mutation has considerable impacts on treatment strategies, vaccination program, diagnosis, and prevention. The present study aimed to detect HBV GENs and mutants in HBsAg-positive patients.

**Methods:**

The study conducted on 4927 patients in Meerut, India, between March 2013 and April 2017. The blood specimens were analyzed for HBsAg using an ELISA kit, then the blood samples from HBsAg-positive patients were subjected to HBeAg assay and DNA isolation. Amplification of the HBV DNA of pre-S gene and pre-core or basal core promoter region were performed by RT-PCR and sequenced to analyze both GEN and mutation.

**Results:**

According to the results, 245 cases were positive for HBsAg, and 55 were HBeAg-positive. With regard to HBV DNA levels, 16 samples were found positive in PCR assay with 7 (43.8%) less than 2000 IU/mL, 4 (25%) between >2000 and 20,000 IU/mL, and 5 (3.25%) >20,000 IU/mL. No mutations were detected in GENs B and A. The prevalence of HBV GENs B and A were 68.8% (n = 11) and 31.25% (n = 6), respectively.

**Conclusion:**

GEN-B was more prevalent in comparison to GEN-A. The genetic diversity of HBV and distribution of its GENs and mutation improve the current knowledge of epidemiological, clinical and virological patterns of hepatitis B in this region, which help physicians to prescribe proper antiviral/interferon therapy according to current genotyping pattern.

## INTRODUCTION

Human hepatitis B virus (HBV) is a small DNA virus inflicting acute and chronic hepatitis. Notwithstanding the availability of a secure and powerful vaccine, HBV infection (HBVI) is, nevertheless, a massive international health hazard. It chronically afflicts about 240 to 300 million humans worldwide, and about 600,000 deaths occur every year due to HBV-related liver pathologies^[^^1^^]^. 

HBV is mainly found in secretions of vagina, semen, blood, saliva, and menstrual blood of infected patients and can be transmitted to the uninfected individuals via contact with the body fluids of infected patients^[^^2^^]^. Meanwhile, in households of chronically infected individuals, HBVI can be transmitted from person-to-person, even through nonsexual contact^[^^3^^]^. The virus is most commonly transmitted vertically in the world^[^^4^^]^. In India, almost 3-4% of the population is infected with HBV, and more than 50% of the cases become chronic. This incidence, seen within the context of India’s huge populace and in the absence of a national immunization program, could trigger an increasing outbreak of infection and liver disease due, in large part, to HBV. Thus, given the government’s apathetic attitude, the HBV epidemiology in India is ended up severely due to the probability that India may emerge as the country with the largest HBVI pool internationally in near future^[^^5^^]^. 

Phylogenetic evaluation has divided the type of HBV into eight genotypes (GENs), defined by using an inter-group divergence of >8% inside the whole genome series^[^^6^^,^^7^^]^ and of >4% inside the S gene^[^^8^^]^. Since the first description of four GENs (A-D) of HBV in 1988^[^^7^^]^, four more have been identified, distinctive E, F, G, and H. Furthermore, sub-GENs with distinctive series traits and a divergence inside the whole genome of >4% have been shown to locate inside GENs A, B, C, and F^[^^9^^,^^10^^]^. The prognosis, initial clinical symptoms, and response to treatment may differ from GEN to GEN in infected patients and there is a variation in geographic distribution of different GENs^[^^11^^]^. The pathogenic variations among HBV GENs have been in part clarified. Intracellular expression ranges of HBV DNA and HBV core antigen (HBcAg), in addition to the extracellular titers of HBV DNA and HBeAg have been observed to be higher for HBV GENs B and C than for A and D. The intracellular accumulation of HBV DNA and viral antigens may additionally play a crucial part in the induction of liver cellular damage. Moreover, the greater replication capability of GEN C may also explain why it is by far the GEN associated with the most extreme HBV-induced liver disease^[^^12^^,^^13^^]^. 

Pre-core mutants are HBV variations that appear for the duration of HBeAg sero-conversion. The most common location of such mutations is a guanine to adenine substitution at nucleotide 1896, creating a premature stop-codon at the pre-core region^[^^14^^]^. The core promoter region is situated upstream to the pre-core region; the regulation of viral replication occurs at this region and consists of an upstream regulatory sequence and a basal core promoter. A1762T and G1764A nucleotide exchange is a mutation that occurs in the core promoter region, resulting in enhanced viral genome replication and substantial reduction in HBeAg expression^[^^15^^]^. The intention of the existing research was to assess the sero-occurrence of HBV as well as detection of HBV GENs and mutants in Meerut, India 

## MATERIALS AND METHODS

**Study background and subjects**

This study was conducted on 4927 patients. Blood samples obtained from suspected HBVIs, and the sequelae patients from Meerut were collected in a clean, sterile and small test tube and processed in the Central Research Station Laboratory of Microbiology at Netaji Subhash Chandra Bose Subharti Medical College (Meerut, India) between March 2013 and April 2017.

**Sample collection and processing**

Ten ml of blood samples was received from patients and analyzed in the laboratory. The sera were separated and screened for HBsAg by Hepa Card (J. Mitra & Co. Pvt. Ltd., New Delhi, India), and positive sera were stored frozen (-20 °C) until tested for viral markers. The HBsAg-positive serum samples were tested for HBsAg using a commercially available ELISA kit (ERBA Transasia Bio-medicals Ltd. Daman, India). The positive serum samples were retested for HBeAg (ELISA; Beijing Kewei Clinical Diagnostic Reagent Inc., Beijing, China). DNA isolation from the serum samples was performed using the QIAamp DNA Mini Kit (Qiagen, Germany) and based on the manufacturer’s recommendations. The isolated DNA was amplified by RGQ real-time PCR using the artus HBV RG PCR Kit (Qiagen, Germany) according to the recommendations provided by the manufacturer. Two sets of primers (two forward and two reverse) were used for both Pre-S gene and Pre-core/core region and one set for detecting the existence of HBV/B in a mixed population. A GEN B-specific PCR using HBV/B GEN-specific primers (PS-B1 and PS-B2) was also performed (Table 1).

**Real-time PCR profile (cycling conditions)**

PCR was carried out in a thermal cycler as follow: an initial denaturation at 95 °C for 10 min and 45 cycles of 95 °C for 15 s, 55 °C for 30 s, and elongation at 72 °C for 15 s.

**Data interpretation and analysis**

**Fig. 1:**
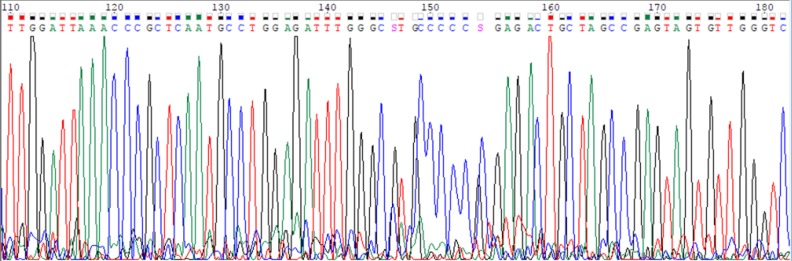
HBV gene sequencing in open gene DNA sequencing system

Data analysis was performed with Rotor Gene Q software (Qiagen, USA) as per the manufacturer’s instructions. After the completion of PCR, the data was acquired on FAM/Sybr (Green) and JOE (Yellow) channels. If the HBV DNA was positive, the signal was detected in fluorescence channel of Cycling Green, and if negative, the signal was detected in fluorescence channel of Cycling Yellow (the internal control). TRUGENE HBV kit was used to genotype and to find out mutation in amplified HBV DNA (https://www. labmedica.com/whitepapers/Trugene_HBV_Assay_Final_cover_8.28.pdf) whose sequencing of Amplified HBV DNA was performed by cross-linking immunoprecipitation method using the same PCR primers. The software assigned the viral GEN and the mutations and polymorphisms present in the sample. The TRUGENE HBV module contains sequences that correspond to the S gene, C gene, polymerase regions for GEN A through H, and a universal mutation reporting reference sequence for comparison. The sequence file was generated by Gene Object^TM^ software (Fig. 1) and was analyzed on NCBI website for HBV genotyping and also analyzed in Bioedit software (http://www.mbio.ncsu.edu/BioEdit/bioedit. html) to find out mutation.

**Table 1:** Real-Time PCR primers for genotyping and mutants

**S.N.**	**Primer**	**Nucleotide sequence (5' to 3')**	**Position (nt)**	**Polarity**
Pre-S gene				
1	PS1	GGGTCACCTTATTCTTGGGA	2814–2833	F
2	PS2	CCCCGCCTGTAACACGAGCA	208–189	R
3	PS3	TTGGGAACAAGATCTACAGC	2828–2847	F
4	PS4	GTCCTGATGCGATGTTCTCC	176–157	R
5	PS-B1	ATTCAAAGCCAACTCAGAAA	2946–2965	F
6	PS-B2	ACAGTATTCTGAGCAGGGCTC	105–85	R

Pre-core/core region				
1	PC1	ACATAAGAGGACTCTTGGAC	1652–1671	F
2	PC2	GAAGGAAAGAAGTCAGAAGGC	1977–1957	R
3	PC3	TACTTCAAAGACTGTGTGTTTA	1704–1725	F
4	PC4	GTCAGAAGGCAAAAAAGAGA	1966–1947	R

F, forward; R, reverse

**Statistical analysis**

Obtained data were analyzed by using the SPSS software for windows version 16. Comparison of data in respect to age groups and gender was performed by Z-test. *p* < 0.05 was considered to be statistically significant.

## RESULTS

**Fig. 2:**
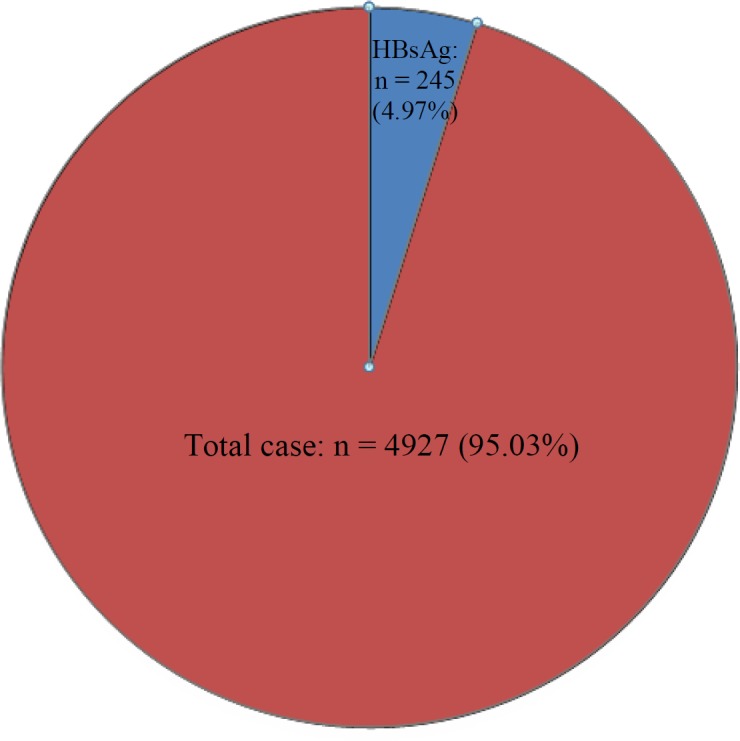
Prevalence of hepatitis B virus

In total, 4927 patients, 2218 (45.01%) males and 2709 (54.98%) females, were included in this study, and those in the range of 21-65 years of age were analyzed for HBsAg. The seroprevalence of overall cases was 4.97% (Fig. 2). Besides, 245 cases were detected as positive and 4682 as negative. Of 4682 negative cases, 2100 were males and 2582 were females.

**Fig. 3:**
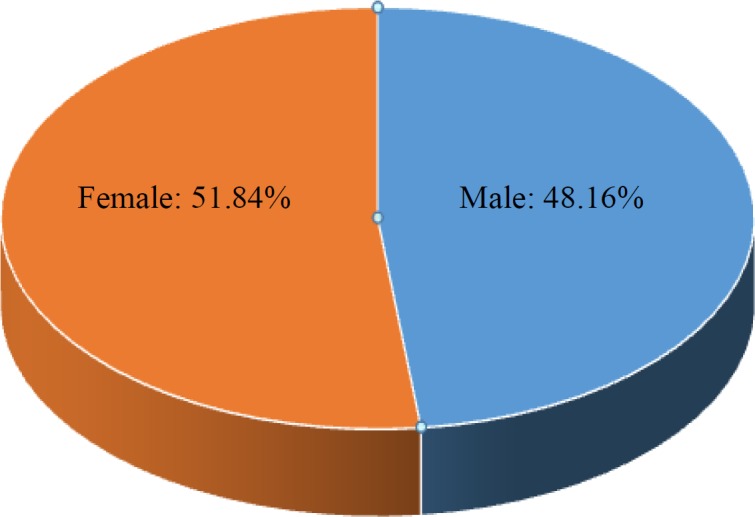
Gender distribution among HBV-positive cases (n = 245).

However, of 245 positive cases, 118 (48.16%) were males and 127 (51.84%) were females, and this was statistically non-significant (*p* > 0.601) by using Z-test (Fig. 3). The sero-occurrence of total males and females were 5.32% and 4.69%, respectively (Table 2).

Of 245 HBsAg-positive cases, 55 were HBeAg positive with a sero-occurrence of 1.12%. Among HBsAg-positive cases, 20 males were positive for HBeAg, and 98 males were negative for HBeAg, and the sero-occurrence of HBeAg-positive males was 36.36%. The highest numbers of HBeAg-positive cases were observed in 21-30-year-old males. Of 55 HBeAg cases, 35 were females, and the rest were non-reactive for HBeAg; all these total female groups were statistically significant (*p* < 0.039) as compared to HBeAg male group by using Z-test, Also, the prevalence of HBeAg-positive females was 63.64% among HBsAg-positive cases. The age group of 21-30 had the highest seroprevalence (49.09%) when compared to other age groups (Table 2). All 245 HBsAg-positive cases were detected for HBV DNA and viral load, except for seven samples that were excluded from PCR due to low volume/spoilage of the samples.

The PCR results showed that 84% (n = 200) of the patients were not detected for HBV DNA, 9.24% (n = 22) were between >threshold (0.05 IU/ml) and<cutoff (10 IU/ml), and 6.72% (n = 16) was cutoff (10 IU/ml). Of these 16 PCR-positive patients, the HBV DNA levels of 7 (43.8%) were below 2000 IU/mL, 4 (25%) were between >2000 IU/mL and 20,000 IU/mL, and 5 (3.25%) were >20000 IU/mL (Table 3). All 16 PCR positive samples were sequenced for HBV genotyping and pre-core and basal core mutants (Table 4). The prevalence of HBV GENB and GEN A were 68.8% (n = 11) and 31.25% (n = 5), respectively. There was no mutation when the pre-core and basal core regions were sequenced.

## DISCUSSION

Hepatitis B, as it so happens, has no seasonal distribution worldwide. Among developed nations, the occurrence is more common in adults, especially sexually active individuals, than in children, as well as in urban than rural areas^[^^1^^,^^2^^]^.In countries like Africa and South America, and some other developing countries, this disease is frequently observed among infants and children and is mostly transmitted vertically, from mother to offspring, or via a close personal contact^[^^1^^,^^2^^]^. However, in endemic areas, most individuals are infected in perinatal stage or in early childhood, and the carrier rates are approximately >5-6%^[^^16^^]^. The rate of carriers is lower in temperate regions than in tropical area and in females than in males^[^^17^^]^. Children born to HBsAg-positive mothers and those who have been fortunate to avoid being infected perinatally continue to be at an excessive risk of infection in the early years of their life. In households, where individuals are chronically infected, non-sexual contact can also transmit HBVI from person-to-person. Transmission happens primarily through the percutaneous path^[^^3^^]^. Other than blood transfusion, some therapeutic, prophylactic and diagnostic methods can also transmit the infection. HBV is extremely infectious and very little quantities of a serum (as low as 0.00001 ml) would suffice for transmitting the disease. Hence, any process that could carry drops of blood/serum from one individual to another can contribute to the transmission of infection^[^^2^^]^. HBV is especially very frequent among narcotics addicts, prostitutes, and male homosexuals. Besides, clinical and paramedical personnel, workers at blood banks, handlers of hemodialysis gadgets, laboratory workers, and personnel working in establishments for the mentally retard individuals could also carry the infection. Outbreaks are known to have occurred among hospital workers and patients^[^^18^^]^. 

In India, the HBV carrier rate is about 3%. Therefore, the severity of HBVI is most certainly not negligible. There are >350 million chronically infected people in the world, and over >1 million die of chronic HBVI (CHBVI) each year. Even though many individuals subsequently gain a state of non-replicative-infection, the prolonged immunologic response to infection results in the formation of liver cirrhosis, even liver failure, or hepatocellular carcinoma (HCC) in up to 40% of the infected individuals^[^^16^^]^. Incidence of HBV varies from nation to nation and is subjected to a complicated interplay of behavioral and environmental and the host’s factors. In general, the incidence is the lowest in countries with high living standards (e.g. the United States, North America, and Europe) and the highest in underdeveloped or developing countries (China, South East Asia, and South America). The situation in India is alarming; almost 3-4% of the population is infected with HBV and CHB, amounting to more than 50% of hepatitis instances. Thus, given the government’s apathetic attitude, the HBV epidemiology in India has become critically relevant to the possibility that India may soon emerge as the country with the largest HBVI pool internationally^[^^5^^]^. The current prevailing facts confirmed 4.97% sero-occurrence much like the research carried out by Rajani and Jais^[^^19^^]^ in New Delhi, which pegged it at 4%. The occurrence of sero-positivity has been discovered to be greater than that suggested in other investigations conducted by Bart *et al.*^[^^20^^]^ (9.5%) in Switzerland and Jain *et al.*^[^^21^^]^ (9.67%) in Lucknow, India. The HBsAg sero-prevalence has also been observed to be lower than that suggested in other researches performed in different areas of India by Das *et al.*^[^^22^^]^ (1.2%) in West Bengal, Khatoon and Jahan^[^^23^^]^ (4%) in Sitapur, Malhotra *et al.*^[^^24^^]^ (1.5 %) in Faridkot (Punjab), Pragati *et al.*^[^^25^^]^ (1.57%) inYavatamal (Maharashtra), and Adisesh *et al.*^[^^26^^] ^(2.8%) in Chennai. Variable outcomes of sero-occurrence for HBV have been found to be 1.55%, 1.43%, 1.09% in different studies in India^[^^22^^,^^27^^,^^28^^]^.

**Table 2:** Sex and Age distribution among HBsAg- and HBeAg-positive patients

**Age group (Y)**	**HBsAg**		**Total (%)**	**HBeAg**		**Total (%)**
	**Male**	**Female**		**Male**	**Female**	
21 - 30	69	74	143 (58.37)	8	19	27 (49.09)
31 – 40	33	29	62 (25.31)	7	13	20 (36.36)
41 - 50	11	15	26 (10.61)	4	3	07 (12.73)
51 - 65	5	09	14 (5.71)	1	-	1 (1.81)
Total (%)	118 (5.32%)	127 (4.69%)	245 (4.97%)	20 (36.36)	35 (63.64)	55 (100)

**Table 3:** Distribution of HBV DNA Viral among HBsAg- and HBeAg-positive patients

**HBsAg** **+ve**	**HBeAg**		**Viral load ( IU/ml)**			
	**- ve**	**+ve**	**NTC threshold**	**>Threshold** **(0.05**)	**cutoff (10)**	**Total**
245	-	55	17	22	16	55
	190	-	183	-	-	183
245	190	55	200	22	16	238

Considering the study conducted by Chattopadhyay^[29]^ in New Delhi India, only GENs A (16%) and D (84%) were found to be associated with chronic liver disease. On delving into a study conducted by Vivekanandan from South India, GENs D, A, and C were detected in 57.3%, 18% and 11.5%, respectively^[^^30^^]^. In an examination conducted by Swati *et al.*^[^^31^^]^ in the western part of India, GENs D and A were most effectively detected; the former was seen to be primary (91.93%), and the latter was observed to occur regularly in 8%. Another study in a post graduate institute at Chandigarh collected GENs D, A, B, and C and in one pattern GEN B and then mixed then with GEN-D^[^^32^^]^. The prevalences of GEN B and GEN A from the isolated HBV DNA were found to be 68.8% and 31.25%, respectively. GEN B was predominant in our isolates, and the variable consequences were discovered in a one-of-a-kind research performed in a part of India^[^^22^^,^^27^^,^^28^^]^.

**Table 4:** HBV DNA viral load showing GENs and mutation

**S.N.**	**Viral load** **(IU/ml)**	**Objectives**				
		**GEN**		**Mutation **	
		**Target regions**	**GenBank No.**	**Target regions**	**Pre-core/basal core**
1	1.00413 × 10^6^	Pre-S gene	B-D00329	Pre-core/core	NM
2	2.75525 × 10^3^	Pre-S gene	B-D00329	Pre-core/core	NS
3	4.23150 × 10^3^	Pre-S gene	A-AF 090842	Pre-core/core	NM
4	2.60000 × 10^1^	Pre-S gene	B-AB073846	Pre-core/core	NS
5	3.78439 × 10^5^	Pre-S gene	A-AF 090842	Pre-core/core	NM
6	5.37500 × 10^1^	Pre-S gene	B-D00329	Pre-core/core	NS
7	7.78550 × 10^3^	Pre-S gene	B-D00329	Pre-core/core	NM
8	5.72500 × 10^1^	Pre-S gene	B-D00329	Pre-core/core	NS
9	3.25000 × 10^2^	Pre-S gene	A-AF 090842	Pre-core/core	NS
10	9.06890 × 10^5^	Pre-S gene	B-D00329	Pre-core/core	NM
11	3.54425 × 10^3^	Pre-S gene	A-AF 090842	Pre-core/core	NM
12	7.36287 × 10^5^	Pre-S gene	B-D00329	Pre-core/core	NM
13	8.39305 × 10^5^	Pre-S gene	A-AF 090842	Pre-core/core	NM
14	4.35000 × 10^1^	Pre-S gene	B-AB073846	Pre-core/core	NS
15	1.00000 × 10^1^	Pre-S gene	B-D00329	Pre-core/core	NS
16	1.12500 × 10^1^	Pre-S gene	B-D00329	Pre-core/core	NS

Eight PCR positive samples were not sequenced for mutation due to low viral load. NM, no mutation; NS, non-sequenceable

Numerous studies carried out outside India have proven the exceptional incidence of GEN C being predominant in Bangladesh and Taiwan^[^^33^^,^^34^^]^, while GEN D is dominant in Turkey and Iran^[^^35^^,^^36^^]^. Predominance of the HBV GEN-E was detected in Niger^[^^37^^]^. The new research works have revealed that acute infection with HBV GEN A could increase the risk of chronic infection. In Japan, instances of HBVI after CHB has been observed to be greater in patients with GEN A (23%) than those with GEN B (11%) or C (7%) infections^[^^38^^].^ Of particular interest is the fact that the increase of acute infections with HBV might bring about a redistribution of HBV GENs among patients with CHB in any country wherein the hepatitis B vaccination has not been released yet. For instance, in a nationwide survey, Matsuura *et al.*^[^^39^^]^ have found that the prevalence of HBV GEN A in CHB sufferers in Japan increased from 1.7% all through 2000 to 3.5% in 2006. GEN F turned into the universal GEN among the CHBVI in the city of Buenos Aires, Argentina, and GEN F showed a propensity to promote an unfavorable disease, thereby resulting in a number of chronic instances^[^^40^^]^. Positive HBV GENs and sub-GENs C, B2-5, and F1 appeared to have a better chance of growing HCC, and other GENs like B1, B6, and A2 seemed to be related to a lower risk of complication of HBV. Those individuals who were infected by GEN C were more likely to develope HCC. Waitlist mortality was the highest amongst patients with GEN D, while incidentally, post-transplant mortality became the highest amongst sufferers with GEN C^[^^41^^]^. In China, HBV co-infections with two or three GENs were related to higher viral-load and development to chronic diseases. HBV B2 infection appears to be associated with HCC reappearance, and HBV C2 was discovered more in HCC patients^[^^42^^]^. In another work from China, it has been demonstrated that HBV GENs B and C are associated with remarkable risk of chronic-stage liver sicknesses, which necessitates transplantation. GEN C also seemed to convey a more hazardous severity of reappearance due to lamivudine-resistant mutants^[^^43^^]^. 

In North India, GEN A is very frequently related with ALT increase, HBeAg-positivity, and absence of anti-HBe, and amongst adults above 25 years, it is more associated with liver cirrhosis than GEN D^[^^44^^]^. Sufferers with HCC and GEN C had greater chances of tumor recurrences after resection of HCC as compared to those with GEN B^[^^45^^]^. In a study conducted in Spain that covered both interferon-treated and untreated pateints, those infected with GEN F had been shown to have decreased cumulative opportunity of sustained biochemical remission and HBV DNA loss and a substantially rates of liver failure than those infected with GEN D or A virus^[^^46^^]^. An observation from India has stated that GEN D is related to greater extreme liver disease and HCC in more youthful sufferers than GEN A^[47]^. In Taiwan, GEN C was associated with more severe liver disease, and GEN B was correlated with the improvement of HCC in young non-cirrhotic sufferers. In comparison, GEN B had a favorably more suitable analysis in Japan and China and was actually related to the improvement of HCC. Similarly, GEN D had a connection with more strong liver disorder than GEN A, which were mostly associated with HCC in younger infected individuals in India^[^^48^^]^. 

In respect to sex-related prevalence, this research showed 5.32% sero-occurrence in males and 4.69% prevalence in females. The high sero-occurrence of HBsAg in males was accountable for the overall high prevalence in all the patients. The basal core promoter dwelling within the overlapping X open reading frame region controls the transcription of each PC and core areas^[^^34^^]^. PC mutations are frequently associated with C gene mutations/deletions. Several types of pc/core mutants had been defined in numerous parts of the world and are seen as being related to increased danger of HCC in addition to the progression of liver diseases. In our research, we have not detected any mutation in surface gene and C gene because this study was prevalence-based research, and this may be the reason why no detection of any mutation transpired in the basal core promoter and PC region. On having a look at the prevailing trend HBV, the sero-occurrence was associated with the relation of age. The positivity for an active carrier turned additionally higher. A higher occurrence of HBsAg detected in this study is presumptively because of the lack of immunization against this virus. The higher occurrence was also directly correlated with the presence of an uneducated populace and unlicensed practitioners, who for numerous decades have taken the advantage of the lack of consumer evidence inside the clinical services arcade and misguided patients to take redundant injections, in developing nations like India. 

The current research shows that the sero-occurrence of HBV was the highest among males. The most predominant HBV sero-prevalence was observed in the age group of 21-30 in both genders, and this may be due to higher sexual activity of this age group. No pre-core and basal core mutants were detected by sequencer, and GEN B was more prevalent in comparison to GEN A.
